# Editorial: Novel treatments and the underlying mechanisms for diabetic foot and related diseases

**DOI:** 10.3389/fendo.2023.1323323

**Published:** 2023-11-20

**Authors:** Johnson Boey

**Affiliations:** Department of Podiatry, National University Hospital Singapore, Singapore, Singapore

**Keywords:** diabetic neuropathy, diabetic foot ulcer (DFU), diabetic limb salvage, mesenchymal stem cell (MSC), amputation

The world is facing an epidemic of diabetes mellitus (DM), in which the International Diabetes Federation (IDF) estimates an increase of 50% to 783 million cases by 2045 (1). The prevalence of DM is higher in those above 75 years of age and in urban cities of middle-incomes countries (1). Approximately 6.7 million people died from diabetes-related complications in 2021 (1). The surge in DM and related complications have been linked to burgeoning global healthcare expenditure, reaching $1 trillion USD in 2021, and it is expected to increase significantly in the next few decades (1).

Diabetic peripheral neuropathy (DPN) is among the common diabetes-related complications that affects approximately 50% of individuals diagnosed with DM (2). Dong et al. found that abnormal vibration perception threshold (VPT) was a significant predictor for altered dynamic gait pattern and stability (P<0.01). In addition, the alteration to the function of the central nervous system have been implicated in the development of DPN. Neuroimaging of the cerebral cortex demonstrated significant changes to the cerebral morphology and function in the early stages of subclinical DPN (Zhao et al.). As DPN progresses, grey matter volume in the orbitofrontal cortex, the region which is involved in emotion and cognitive processing such as decision-making, decreases (Zhao et al.). The loss of protective sensation and vibration perception due to DPN predisposes diabetic foot ulcerations (DFUs) (3). Effective offloading modalities are essential to the management and prevention of DFUs. Hemler et al. designed specialized footwear with automated conformable insoles that actively adapt to patients’ feet according to their plantar pressure distribution map. Further clinical trials will be necessary to evaluate the usefulness of this product.

It has been accepted that there is a 25% lifetime risk of developing DFUs (3). The prognosis of diabetes-related foot complication are often grim, and the 5-year mortality rate is comparable to that of cancer (4). The pathophysiology of DFUs is multi-factorial and it is believed that structural foot deformity and sensorimotor deficit give rise to elevated plantar pressure which lead to foot ulceration. A nomogram model for the prediction of DFUs in elderly patients validated that age, DPN, lactate dehydrogenase, high-density cholesterol, total serum cholesterol, and smoking strongly influenced the development of DFUs (Shao et al.). Patients with DFUs require close monitoring and appropriate wound care management by multi-disciplinary foot care teams. The emergence of telemedicine and digital therapeutics has revolutionized remote patient monitoring, which has benefited both the patient and clinician during the recent COVID-19 pandemic. Keegan et al. observed an average reduction of 41.6% in wound area (p=0.005) and wound healing rate of 12% (3/25) through the use of a smartphone application.

The persistent inflammatory response exacerbates the release proteases and reactive oxygen species (ROS) that culminate in the impairment of fibroblast, growth factors, and extracellular matrix proteins (Zhu et al.). As a result, DFUs often result in lower extremity amputation (LEA) due to infection with or without a background of peripheral arterial disease (PAD) and/or infection. Laboratory investigation in patients with DM who had undergone LEA revealed reduced levels of serum albumin and poor ankle-brachial index (ABI) but elevated inflammatory biomarkers, including white blood cells (WBC), C-reactive protein (CRP), and fibrinogen (Gong et al.). Among the risk factors explored, a history of previous amputation (OR 10.194; P=0.001), gangrene (OR 6.466; P=0.010), and ABI (OR 0.791; P= 0.032) were significantly associated with LEA. The management of osteomyelitis revolves around effective source control using treatment options varying between antibiotic regimens and surgical resection of infected areas. The outcome of pharmacotherapy depends on of appropriate culture-directed antibiotic regimens. In a retrospective study by Xu et al., the authors revealed that specimens taken from DFO grew predominately gram-negative bacilli but there were low concordance rates (19.3%) between deep tissue and bone specimens. Nonetheless, only 15.7% (9/57) of specimens were negative in both pathology and bacterial culture after conservative debridement. This group of nine patients healed significantly faster than those with residual infection after conservative debridement.

The concept of angiosome theory has been used widely in diabetic limb salvage. Similar to dermatome, the perfusion of an area of soft tissue, known as an angiosome, is provided by source arteries, but a single source artery can perfuse multiple overlapping angiosomes along its path (5). This principle is demonstrated in a case report of a non-healing posterior heel ulcer complicated by osteomyelitis and extensive PAD (Chen et al.). Balloon angioplasty was performed in all primary source arteries except the posterior tibial artery (PTA). In this case, the peroneal artery (choke vessel) was able to provide perfusion to the posterior heel despite occlusion in the PTA (primary source artery). Therefore, these choke vessels function as a compensatory safety mechanism. Secondly, tibial transverse transport (TTT) has been performed increasingly in the recent decades for the treatment of DFUs with advanced PAD, owing their ability to improve local vascularity and stimulate cellular proliferation (Hu et al.). In particular, the healing and limb salvage rate after TTT is 0.96 and 0.98, respectively. TTT significantly improved wound healing (OR: 10.43; p< 0.001) and limb salvage rates (OR: 9.65; p>0.001) when compared with a control group (Hu et al.). The common complications encountered with TTT were pin-associated infection (8%), DFU recurrence (2.9%), and fracture at the transportation site (2%). Nonetheless, there remains much heterogenicity in the clinical protocol of TTT. Another promising adjunct treatment for the management of DFUs is the use of mesenchymal stem cells (MSC). The potential of MSC lies in their pluripotent ability, secreting growth factors and cytokines that stimulate the proliferation and migration of fibroblast and endothelial cells and promoting angiogenesis (Yu et al.). In addition, MSCs have also been found to exert immunomodulatory effects by inducing macrophages to express anti-inflammatory M2 phenotype (Yu et al.). Despite these cellular effects, the variable clinical efficacy from the heterogeneity of MSCs poses considerable challenge to formulating a treatment protocol appropriate for its clinical application (Yu et al.).

In this Research Topic, the authors illustrated the global efforts to deepen our understanding of diabetes-related foot disease and the relentless search for novel treatment modalities. With globalization, the changes in lifestyle, dietary patterns, and physical activity is driving an uptrend in chronic diseases such as obesity and DM. The focus in our fight against DM goes beyond treatment but prevention through education. It takes more than an individual and community to agree upon the strategy to mark our path towards a diabetes-free world. It would be just be an imagination if we all don’t act now.

## Author contributions

JB: Writing – original draft, Writing – review & editing.
